# The Role of Habits and Motivation in Human Drug Addiction: A Reflection

**DOI:** 10.3389/fpsyt.2014.00008

**Published:** 2014-01-29

**Authors:** Zsuzsika Sjoerds, Judy Luigjes, Wim van den Brink, Damiaan Denys, Murat Yücel

**Affiliations:** ^1^Department of Psychiatry, VU University Medical Center, Amsterdam, Netherlands; ^2^Department of Psychiatry, Academic Medical Center, University of Amsterdam, Amsterdam, Netherlands; ^3^Brain Imaging Center, Academic Medical Center, University of Amsterdam, Amsterdam, Netherlands; ^4^Max Planck Institute for Human Cognitive and Brain Sciences, Leipzig, Germany; ^5^Netherlands Institute for Neuroscience, Institute of the Royal Netherlands Academy of Arts and Sciences, Amsterdam, Netherlands; ^6^Monash Clinical and Imaging Neuroscience, Monash Biomedical Imaging Facility, School of Psychological Sciences, Monash University, Melbourne, VIC, Australia

**Keywords:** habits, habit formation, motivation, addiction, goal-directed behavior

## Introduction

This Research Topic in Addictive Disorders and Behavioral Dyscontrol, a section of the journal Frontiers in Psychiatry, focuses on motivational mechanisms underlying substance use, abuse, and dependence. This is an important topic in addiction research, since most psychobiological models of drug addiction consider the motivational or reinforcing aspects of drugs to be the central drive for drug use [for an extensive overview of craving and motivation-based addiction models, see a review by Skinner and Aubin (1)]. However, motivational models alone do not seem to fully cover the complexity of addictive behaviors observed in humans, especially in relation to the more chronic, highly relapsing patterns of addiction. In recent years, habit formation theory has become more prominent for explaining the persistent pattern of addiction despite decreasing reinforcing properties of the drug and increasing negative consequences of continued drug use. According to this model, there is a shift from motivated goal-directed behavior toward more automatic and habitual behavior over the course of long-term drug abuse, which is extensively described by Everitt and Robbins (2–4). Within this framework, which is derived primarily from animal studies, habits and goal-directed behaviors (the latter being behavior motivated by the desirability of the goal) are opposing ends of the spectrum. However, human behavior is more complex than observed in laboratory animal settings, as is confirmed by clinical observations, and translation from animal to human behavior remains a challenge. Moreover, motivations and habits could be more intertwined than previously assumed. Therefore, some questions rise considering the construct of habits: is habitual behavior completely devoid of motivational underpinnings (i.e., goal-directedness) or is it possible that motivation still plays a role in habitual behavior? Moreover, is habit a unitary construct or are there different types of habituation? In this article, we present considerations in the context of human addiction and motivation in order to open the discussion toward a more careful consideration of the concept of habit and its role in drug addiction.

## Motivation

In most motivational models of addiction, positive reinforcements (or rewards) are highlighted as the initial and primary drive for drug use. To this end, the incentive sensitization model by Robinson and Berridge (5) states that drug cues can attain incentive saliency when repeated exposure to drugs and drug-related cues (such as drug paraphernalia) enhances the memory of the anticipated reward. In other cases, negative reinforcement gains importance when drug intake is reinforced by the avoidance of aversive consequences induced by drug withdrawal, as described by Solomon and colleagues as the opponent-process theory of motivation (6, 7), as well as the concept of “allostatic load,” described by Koob and colleagues in classical avoidance theories (8–11). Together, by including (positive or negative) reinforcement as a central drive for drug abuse, these motivational addiction models imply a high level of goal-directedness.

## Habits

Animal lesion and devaluation studies, however, show that over the course of progressive drug use, reinforcing properties of drugs lose their value, and goal-directedness decreases. With decreasing goal-directedness over time, regard for associated outcomes decreases, and drug use behavior is progressively driven by drug-related stimuli only (12–15). This stimulus-driven behavior is often described as habitual behavior and in the context of a drug-taking “habit” it has been operationalized as behavior that has become automatized, highly stimulus bound, inflexible, and insensitive to the associated outcomes (positive or negative) (2, 3, 16). It is the habit formation model, primarily based on rodent studies, that describes this shift from goal-directed toward habitual drug use behavior. This idea of decreasing relevance of reinforcing effects is not new. More than 50 years ago, Chein and colleagues (17) already questioned the notion of addiction as a sole consequence of rewarded behavior. They showed that a large proportion of healthy individuals, who had consumed various drugs, found the effects pleasurable; however, they did not go on to become dependent drug users. Furthermore, they showed that a small percentage of those who were dependent found the initial drug experience unpleasant, but nevertheless went on to become chronic users. Accordingly, the role of craving in addiction does not seem to have a one-on-one relationship with consumption, since craving without consumption can occur, but more importantly, consumption without craving can also occur. Since in motivational drug use models craving plays a major role within a larger goal-directed decision-making framework (18–20), consumption without an internal motivational drive such as craving is not covered by these motivational models of addiction. In other words, the initial importance of the rewarding properties of drugs as is emphasized by several motivational models of addiction does not always appear to be the key drive of ongoing drug use. Therefore, motivation models, although very adequate on many occasions, do not cover all aspects of drug use, especially when it comes to long-term chronic dependence. Applying the habit formation model, albeit in a more refined manner, could offer a solution here.

Although habit formation seems to be a very well suited model to cover aspects of drug addiction that are not enclosed in motivational models, some further considerations are required in order to improve the translation of this animal-based model to the human equivalent of addictive behavior. The seemingly simple stimulus–response contingencies described in the habit formation model are mainly based on experiments with lever-pressing rats. However, in its current form, the habit formation model represents habitual behavior as a singular construct opposing goal-directed behavior, which may be too simplistic for encompassing more complex human habitual patterns, which may be intertwined with motivational drives in some occasions. Note that we do not question the increasing role of habituation in addiction outlined by this model and do stress the important contribution it has made to our understanding of chronic relapsing drug use. In fact, some recent human neurobiological studies also indicate the presence of habit formation and its associated neural shift from ventral to dorsolateral striatum in chronic drug dependent patients (21, 22). However, the question is whether complex human behavior can be sufficiently explained by simple stimulus–response actions as described in the current habit formation model. Perhaps a next step involving a more nuanced concept of habituation may improve the model for translation to human behavior.

## Different Types of Habit

Patient self-reports confirm that rewarding or pleasurable properties of drugs play a decreasing role over the course of long-term addiction (23). After repeating the same behavior (or sequence of behaviors) over many years, habituation undeniably becomes more relevant, and stronger associations develop between various stimuli and linked responses. However, there may be different types of habituation: for example, imagine that a patient who has been abstinent for months is visiting an old and familiar drinking companion. He has been in the house of his friend where they used to drink many times before. The patient suddenly finds himself walking to the fridge of his friend’s kitchen to take a beer. This behavior seems to be based on a stimulus (S) – response (R) contingency (S: house of friend; R: taking and drinking a beer). The behavior may be the result of a “simple” (or motor) habit without the co-occurrence of any specific thought, feeling or urge (such as subjective craving), in the same way other people may automatically wash their hands after going to the bathroom. In these types of habits, the role of goal-directedness at the time of performing the behavior is likely to be absent. Alternatively, the (habitual) behavior could also be the result of an underlying (motivational) urge such as increased craving elicited by the environment of his friend’s house in which he used to drink. In this latter scenario, craving modulates the S–R connection. One could argue that when craving modulates the S–R connection, the drinking can still be a “habitual” response to craving. However, in contrast to a simple S–R contingency, behavior that is modulated by craving or any other emotional/motivational state seems at least partly goal-directed (i.e., to reduce craving). In this situation, outcome devaluation (i.e., craving reduction) is expected to influence the behavior, and therefore the behavior would not be habitual according to the dichotomy of goal-directed versus habitual behavior (24). Nevertheless, the stimulus-driven and repetitive nature of this reaction to an emotional or motivational state may result in a behavioral sequence that resembles “simple” S–R (motor) habits in other aspects: in both cases with repetition over time less alternative responses seem available and the behavior becomes less flexible and more persistent. It becomes more and more difficult to adapt the behavior, even if it leads to undesirable consequences. As behavior that leads to craving-relief can be considered a form of positive reinforcement, negative reinforcement is also an important motivational drive underlying addictive behavior. Compulsive behavior refers to a repeated response pattern to a negative emotional state (e.g., tension, anxiety, or withdrawal) leading to undesirable long-term consequences (Luigjes and colleagues, in preparation). Compulsivity has been defined as the urge to carry out the act; in the experience of the individual that particular act “has to” be performed (25). Contrarily, simple S–R (motor) habits are characterized by direct S–R contingencies without modulation by urges, thoughts or feelings, but are rather driven by direct motor-schemes. In the literature, the concept of habit is frequently used interchangeably or in combination with compulsivity (e.g., “addiction as a maladaptive compulsive habit” or “compulsive drug seeking”) to indicate a persistent use of drugs in the face of negative consequences (26). It is our view that compulsive behavior can be related to motivational habits, but that it is distinct from motor habits. Yet, both compulsivity and motor habits are likely to play a role in addiction. Other motivational mechanisms related to persistent drug use, such as attentional bias and approach bias, may also drive the onset of habitual behavior in a less conscious and more implicit manner, and could therefore also contribute to motivational habits as proposed before.

## Conclusion

In conclusion, habits have been studied in animals and healthy volunteers [see Table 1 for examples of paradigms applied in (healthy) human studies on habits, and for a recent, more complete overview, see Ref. (27)], but very little is known about pathological/maladaptive forms of habit formation such as in addiction, and currently no gold standard for the study of habits in addictive disorders is available. Addiction is a multi-faceted and complicated series of processes, and in clinical practice simple S–R contingencies and S–R connections modulated by motivational states may often co-occur and may be difficult to disentangle from each other. Nevertheless, in our scientific endeavors, we need to better understand addiction and deconstruct its constituent and complex behavioral expressions into more precise and generalizable scientific models. To this end, we need to be more precise in classifying behaviors and develop a more refined definition of habit formation. It may be an oversimplification to only think of habitual addictive behavior as “simple” S–R contingencies. Moreover, even in non-addictive habitual behavior, goal-directedness and volition may still have some relevance. If both simple S–R contingencies and repetitive response patterns to emotional/motivational states are referred to as habits (which is the case for e.g., compulsive behavior) it may be useful to distinguish different kinds of habits: motivational habits (comprising an emotional/compulsive drive) versus motor habits. In order to improve our understanding of these processes, a more comprehensive investigation of habitual addictive behaviors is warranted in order to determine the underlying mechanisms and possible differences between patients with an addiction and patients with other disorders also associated with compulsive or habitual behavior, such as patients with an obsessive–compulsive disorder and patients with impulse control disorders (28). Both motivational and motor types of habituation may take a more prominent role in regulating drug use during the progression from early to chronic forms of addiction (2, 3, 29). Moreover, motor habits that directly result from stimuli in the environment may need a different therapeutic approach than habits resulting from a reactive pattern to motivational/emotional states. We expect, as a result of these developments in research, that staging and profiling will become paramount in future treatment of addiction (30). As a next step in addiction research, we recommend the development of a more refined conceptualization and improved measurement of habits in addictive behavior based on a closer examination of the potential motivational underpinnings associated with habitual, automatic patterns in long-term drug addiction.

**Table 1:** **Examples of paradigms used to measure habit-related constructs in humans**.

Paradigm	Source/first use	Short description of the paradigm	Selection of studies in humans using this paradigm
Probabilistic classification task, e.g., “Weather prediction task”	Gluck and Bower (31)	Subjects gradually learn to classify stimuli into two categories, based on trial-by-trial feedback. Because of the probabilistic structure of the task, the normal tendency to try to memorize a solution is defeated, and therefore subjects can learn without the use of declarative memory. Since the most useful information is acquired across many trials, the task is proposed to involve gradually acquired habit learning	Knowlton et al. (32), Knowlton et al. (33), Foerde et al. (34)
Discrimination learning task	Bayley et al. (35)	A declarative memory task where habit memory is proposed to involve slowly acquired associations between stimuli and responses that develop outside awareness and are rigidly organized, with the result that what is learned is not readily expressed except when the task is presented just as it was during training	n.a.^#^
Instrumental conditioning task	Valentin et al. (36)	Overtraining on a probabilistic instrumental learning task (choosing between drinks), followed by devaluation (selective satiation on one drink)	Schwabe and Wolf (37), Schwabe et al. (38), Hogarth et al. (39)
Free operant task	Tricomi et al. (40)	Subjects are either given little training, or are over trained on instrumental responding, with a rewarding outcome delivered on a variable-interval reinforcement schedule. This is followed by outcome devaluation and an extinction test	n.a.^#^
Instrumental learning task “Fabulous fruit game”	De Wit et al. (41)	Instrumental learning based on either stimulus–response-outcome (goal-directed) contingencies or direct stimulus–response (habitual) learning induced by conflict. Followed by an instructed outcome devaluation test, and a “slips of action” test, measuring habitual tendencies	Gillan et al. (42)*, De Wit et al. (43), Sjoerds et al. (21)**
Markov decision task	Daw et al. (44)	Applied in the computational neuroscience framework, based on the reinforcement learning theory. A forced choice task that can be solved by using either model-free (inflexible, computationally efficient, habit-like), or model-based (forward planning, flexible, goal-directed-like) control	Gläscher et al. (45), Wunderlich et al. (46), Smittenaar et al. (47)
Shock avoidance learning	Gillan et al. (48)*	Inducing habits by overtraining on avoiding electric shocks, followed by an instructed outcome devaluation phase to test for the level of habit formation	n.a.^#^

*We do not aim to provide a comprehensive overview of methods for habit measurement. For a more complete overview of frequently used paradigms in the current habit literature, we refer the reader to the recent review by Dolan and Dayan (27)*.

*^#^To the best of our knowledge there have not been other studies using this paradigm to measure habit-related constructs*.

*Applied in an obsessive–compulsive disorder (OCD) population.

**Applied in an addiction population (alcohol dependence patients).
